# Analytic methodology for childhood predictor analyses for wave 1 of the Global Flourishing Study

**DOI:** 10.1186/s44263-025-00142-0

**Published:** 2025-04-30

**Authors:** R. Noah Padgett, Matt Bradshaw, Ying Chen, Richard G. Cowden, Sung Joon Jang, Eric S. Kim, Koichiro Shiba, Byron R. Johnson, Tyler J. VanderWeele

**Affiliations:** 1 Department of Epidemiology, Harvard T.H. Chan School of Public Health, Boston, MA USA; 2 Human Flourishing Program, Harvard University, Cambridge, MA USA; 3 Baylor University, Institute for Studies of Religion, Waco, TX USA; 4 Department of Psychology, The University of British Columbia, Vancouver, Canada; 5 Department of Epidemiology, School of Public Health, Boston University, Boston, MA USA; 6 Department of Sociology, Institute for Studies of Religion, Baylor University, Waco, TX USA; 7 School of Public Policy, Pepperdine University, Malibu, CA USA; 8 Department of Biostatistics, Harvard T.H. Chan School of Public Health, Boston, MA United States

**Keywords:** Global flourishing study, Methodology, Childhood predictors, Meta-analysis, Mastery

## Abstract

In this article, we describe the statistical and design methodology of the demographic variation analyses used as part of a coordinated set of manuscripts for wave 1 of the Global Flourishing Study (GFS). Aspects covered include the following: childhood predictors regression analyses, accounting for the complex sampling design, missing data and imputation, sensitivity analysis for unmeasured confounding and meta-analysis. We provide a brief illustrative example of the childhood predictor analyses using the sense of mastery construct indicator from the GFS survey and conclude by outlining some strengths and limitations of the methodology employed.

**Supplementary Information:**

The online version contains supplementary material available at 10.1186/s44263-025-00142-0.

## Background

The Global Flourishing Study (GFS) is a large, multinational panel study that aims to explore the distribution, determinants, and interrelations of various concepts related to human well-being with more than 200,000 people across a geographically and culturally diverse set of countries around the world [1–3]. Interest in flourishing has surged in recent years across various fields like psychology, economics, and public health [4–10]. However, many aspects of well-being remain underexplored, especially globally, as much of the well-being literature has been shaped by Western perspectives [7, 11]. As a multinational panel study, the GFS provides an avenue to explore well-being and flourishing from a multicultural perspective to fill this gap.

The purpose of this article is to describe the methodology applied to the set of construct-specific *childhood predictor* analyses that were produced using currently available wave 1 data from the GFS, most of which are planned for inclusion in manuscripts that are being considered for publication as a coordinated set of manuscripts on wave 1 of the GFS. These childhood predictor analyses apply a common preregistration template principally focused on exploring the distribution of the associations that various childhood experiences have with scores on indicators of aspects of flourishing in each country and how the associations vary across countries.

Evaluating the associations of childhood factors with present aspects of flourishing provides a valuable contribution to understanding the determinants of flourishing as shaped by experiences growing up. Retrospective childhood experiences evaluated include quality of relationship with mother, quality relationship with father, parental marital status growing up, subjective financial status of family growing up, self-reported history of abuse during childhood, feeling like an outsider in one’s family when growing up, and self-rated health growing up. Many of the constructs included in the GFS are seldom included in cross-cultural cohort studies (see survey development report [12]), providing a unique opportunity to strengthen existing knowledge about aspects of well-being from a multinational perspective. All analyses are conducted separately by country, which not only preserves potential heterogeneity in the interpretation of survey items across countries but allows the results to be contextualized considering the sociocultural particularities within each country. Then, country-specific results are pooled using meta-analytic techniques to summarize the associations of childhood experiences with present aspects of flourishing. These analyses provide a template for evaluating the determinants of flourishing globally, and the use of a consistent methodology across manuscripts allows for comparability of results across many different aspects of flourishing.

There are three core components of the current article. First, we begin by providing a high-level description of the data and measures used in the childhood predictor analyses. Next, we discuss aspects of the methodology, namely the regression analyses, accounting for the complex sampling design, missing data and imputation, and meta-analysis. Lastly, we use the sense of mastery outcome (*How often do you feel very capable in most things you do in life?*; response options include always, often, rarely, never) to provide an illustrative example of the analyses and results that will be presented in the construct-specific childhood predictors analyses manuscripts; see Kim et al. [13] for more details on the mastery outcome.

### Global flourishing study data

Currently available wave 1 GFS data includes nationally representative samples of the adult population (18 years old and older) from 22 geographically and culturally diverse countries, including Argentina, Australia, Brazil, Egypt, Germany, Hong Kong (Special Administrative Region of China), India, Indonesia, Israel, Japan, Kenya, Mexico, Nigeria, the Philippines, Poland, South Africa, Spain, Sweden, Tanzania, Turkey, the UK, and the USA (wave 1 data will also become available for mainland China once wave 2 data are released in early 2025). These countries were selected to (a) maximize coverage of the world’s population; (b) ensure geographic, cultural, and religious diversity; and (c) prioritize feasibility and existing data collection infrastructure. The study encompasses approximately 64% of the global population. These countries also include some of the world’s largest and most influential communities of religious believers, including Christians (Nigeria, Brazil, Germany, Philippines, South Africa, USA), Muslims (Indonesia, Nigeria, Turkey, Egypt), Buddhists (Japan), Hindus (India), and Jews (Israel). Data collection was carried out by Gallup, a global analytics and advisory organization with decades of experience collecting global data on various aspects of human life. Most of the data for wave 1 were collected in 2023, with some countries beginning data collection in 2022; exact dates of data collection vary by country [14]. The GFS is set to continue with four additional waves of annual panel data collection from 2024 to 2027. The precise sampling design that was used to collect wave 1 data varied by country to ensure nationally representative samples for each country. Further details of the sampling design methodology are available elsewhere [14, 15].

Survey items included numerous aspects of well-being such as happiness and life satisfaction, physical and mental health, meaning and purpose, character and virtue, close social relationships, and financial and material stability [16], along with numerous other demographic, social, economic, political, religious, personality, childhood, community, health, and well-being variables. Development of the GFS survey occurred over eight distinct phases: (1) selection of core well-being and demographic questions; (2) solicitation of social, political, psychological, and demographic questions from domain experts worldwide; (3) revision of the initial survey draft based on feedback from scholars around the world representing various academic disciplines; (4) modification of question items following input from experts in multinational, multiregional, and multicultural survey research; (5) survey draft refinement based on compiled input from an open invitation to comment, posted publicly, and sent to numerous listservs; (6) questionnaire optimization with support from Gallup survey design specialists; (7) adaptation of items from an interviewer-administered to a self-administered survey instrument using best practices for web survey design to minimize item non-response, illogical responses, and incomplete responses; and (8) confirmation by scholars in several participating countries that translations accurately captured the intended meaning of each question [3, 15].

The data are publicly available through the Center for Open Science (https://www.cos.io/gfs). During the translation process, Gallup adhered to TRAPD model (translation, review, adjudication, pretesting, and documentation) for cross-cultural survey research [17]. Additional information about methodology and survey development can be found in the GFS Questionnaire Development Report [3, 7], as well as the GFS Methodology [14], GFS Codebook (https://osf.io/cg76b), and GFS Translations documents [18].

### Measures

#### Childhood predictor variables

A total of 17 childhood predictors were initially considered and preregistered. We initially preregistered to use all (17) retrospective recall items about childhood characteristics that were included in the GFS intake survey. The survey development process included multiple phases, the details of which are described in Lomas et al. [7]. The 17 items included year of birth/age, gender, race/ethnicity, immigration status, childhood abuse, feeling like an outsider in family, childhood health, subjective financial status growing up, parental marital status growing up, relationship with mother during childhood, relationship with father during childhood, feeling loved from mother growing up, feeling loved from father growing up, religious affiliation growing up, religious service attendance growing up, religious service attendance of mother growing up, and religious service attendance of father growing up. Religious affiliation was assessed in all countries, but the observed response options varied greatly across countries. Racial/ethnic identity was assessed in some but not all countries, and response options were unique to each country to be locally relevant. Additional details about the items and response options for each are reported in the GFS Questionnaire Development Report [3] and the GFS Codebook (https://osf.io/cg76b).

In general, recorded responses of “don’t know,” “refused,” “skipped,” “prefer not to answer,” and “does not apply” were coded as missing. However, the parental relationship predictors were re-coded with an additional indicator such that if a recorded response was “does not apply” to any of the parent relationship items, a 1 was used to refer to such cases and this control indicator was 0 otherwise. Responses to a parental relationship variable may be “does not apply” because either parent has died or was not present for some other reason, which would be an important childhood event to control for in the analysis. We used this additional coded indicator to control for such possible childhood events in the country-specific regression analyses.

The entire set of childhood predictors that were included in the initial preregistration could not be used due to issues with multicollinearity encountered during preliminary testing of statistical analysis code. This multi-collinearity was evident in the strong correlations of certain variables with other; in very large standard error estimates for these variables in the regression models; and in distorted heterogeneity estimates in the meta-analysis (see below). As a result of this multicollinearity, a reduced set of 13 childhood predictors was used. The removed predictors were (1) feeling loved from mother, (2) feeling loved from father, (3) religious service attendance of mother, and (4) religious service attendance of father. The two relationship quality variables with mother and father were strongly correlated with the love from mother and father variables. In consultation with partners at Gallup, the loved from parent items were dropped from the regression analyses because the relationship items were generally interpreted with greater consistency, and subject to fewer translational issues, across countries and languages. The parental religious service attendance while growing up items were especially collinear with the individual’s self-reported religious service attendance while growing, prompting the removal of the former from the set of predictors. We comment on multicollinearity in more detail below.

#### A note on multicollinearity

Although we expected that at least some of the childhood predictors would be correlated, we anticipated that the effects of multicollinearity would be minimal because there are important conceptual distinctions between even those predictors we might expect to be more closely related (e.g., general quality of relation with father vs. feeling love from father). We also anticipated that any concerns about multicollinearity might be mitigated by large country sample sizes. This was the case in some countries, such as the USA (*N* = 38,312), where the full set of initially preregistered childhood predictors was estimable with reasonable standard errors. However, we encountered multicollinearity issues for a number of outcomes in several countries, particularly when sample sizes were smaller. For example, in the Turkey sample (*N* = 1473), coefficient estimates when using the full set of childhood predictors could not be obtained with reasonable standard errors. Our appendix provides the country-specific results for Turkey and the USA after regressing sense of mastery on the full set of predictors for these two samples (see Additional File 1: Table S1) for illustration. Additional File 1: Table S1 also includes the meta-analysis results and the estimate of heterogeneity for each effect.

To try and address concerns about multicollinearity, we considered using a regularizing estimator such as the LASSO [19]. However, in consultation with colleagues at the Institute for Quantitative Social Science at Harvard University, there were not widely accepted approaches to incorporating complex sampling design adjustments or missing data into existing regularizing estimators, nor could we find an approach that was integrated into all software packages that might be used to perform the analyses. Thus, a decision was made to update and document a modification of the preregistration template by removing the four abovementioned childhood predictors from all analyses. Decomposing the effects of such highly related but conceptually distinct aspects of the childhood experience will be left to subsequent investigations where a more nuanced approach to statistical methodology can be considered.

#### Outcome variables

A range of continuous, binary, Likert-type, and nominal response scales were used to assess the different constructs included in the GFS. All items with at least 10 ordered responses were treated as approximately continuous. All remaining Likert-type and nominal items were recoded into binary variables based on cutoffs specified in the preregistrations for each outcome.

## Childhood predictors analyses

This section describes the analyses which were carried out within each country, and we later describe the random effects meta-analyses used to summarize results across countries. Analyses were implemented across multiple software packages (R [20], Stata [21], SAS [22], and SPSS [23]) to ensure consistency in results and ease of use by the larger core group [24]. Implementing the analyses in separate software allows for a greater reach across fields for others to utilize and replicate our analyses in their software of choice. Any deviations across software packages implementation are described below.

### Separating analyses by country

The core analyses of the GFS were conducted separately within each country. As described below, summary statistics were obtained by random effects meta-analysis rather than for example by use of a multi-level model. A key advantage of this approach is that it does not presume cross-cultural measurement equivalence of the measures, which is important because most constructs were assessed using a single item, and cognitive testing during the survey development process suggested some variation in the interpretation of items across countries [25, 26]. Thus, it may be preferable to treat the measures as closely related, but not identical, assessments of each construct across the countries. We chose to conduct analyses separately for each country because it preserves potential heterogeneity in the interpretation of survey items across countries, and it allows the results to be contextualized in light of the sociocultural particularities of each country. This approach also aligns with our decision to use a random effects meta-analysis to combine effect estimates for each childhood predictor category, which implies that the regression coefficients we combine are not necessarily representative of effects estimated from repeated samples from the same population of respondents but the effects vary across countries forming distinct populations with on those effects. The resulting meta-analyzed effects represent the average effect across populations, but without assumptions of the equivalence of those effects on different populations.

### Regression analyses

Manuscripts that apply the preregistration template for the construct-specific childhood predictor analyses used complex survey-adjusted regression models to evaluate the effect of coded childhood characteristics on the GFS outcome. The type of regression model depended on the scale of the outcome, but the general form of the model is:

$$y=f(\text{X}\beta )+e$$

where $f\left(.\right)$ is the link function which is either the identity function for continuous outcomes or the exponential function for modified Poisson regression; $\text{X}$ is the design matrix of childhood predictors; $\beta$ is the vector of regression coefficients; and $e$ is the vector of errors distributed according to the outcome.

When the outcome is binary, we conducted modified Poisson regressions. Modified Poisson is a popular approach to estimating risk ratios [27]. The exponentiated coefficients from the results of the modified Poisson can be interpreted as risk ratios: a risk ratio is the ratio of the probability of an outcome in an exposed group to the probability of an outcome in an unexposed group. Logistic regression was not used for binary outcomes because the resulting odds ratio estimates are not infrequently incorrectly interpreted as risk ratios. This is especially problematic and inaccurate when binary outcomes are common (prevalence between 0.10 and 0.90), as is the case for many outcomes in the GFS. To aid in comparability of results across outcomes, and to avoid these problems with interpretation, risk ratios obtained by modified Poisson regression were employed throughout. However, risk ratios are non-invariant under re-labeling of the outcome categories (and readers should not misinterpret these as odds ratios).

Point estimates of effects were obtained using weighted least squares. Robust standard errors were computed using a Taylor series linearization approach and adjusted for stratified sampling when necessary. However, each software package differs in how these methods are implemented, resulting in minor variations in results across packages.

For analyses conducted using R, the *survey* package [28] was used to estimate the model. For analyses conducted in Stata, the built-in *svy: reg* and *svy: poisson* procedures were used [29]. For analyses through SAS, there was no built-in functionality for estimating a complex survey adjusted modified Poisson model. We modified a macro, *%surveygenmod*, [30] published as a conference proceeding of the SAS Global Forum in 2017 to obtain the necessary functionality in SAS; we needed to make several significant changes to the original macro, culminating in the modified macro *surveygenmod2* [31].

#### Complete separation

For categorical outcomes, an issue known as complete separation [32] can sometimes occur when estimating the modified Poisson regression model. Complete separation concerns cases in which all respondents in one level of a childhood predictor variable have the same value on the outcome. This results in uninterpretable estimates for those effects, as there were no observed differences between the childhood predictors and the outcome. We have tried to note when this occurs in the country-specific results and the effect that such cases might have on the results of the meta-analyses.

#### Joint test of effects for groups of coefficients

We used Wald tests to conduct a joint hypothesis test of whether a group of parameters are significantly different than zero. A Wald-type test was the most straightforward approach to implement in different software to obtain comparable results and *p*-values. The general form of the Wald-type tests implemented across software is as follows, where the estimated parameter variance–covariance matrix ($\widehat{\text{V}}$) to test the hypothesis ${H}_{0}: \text{L}\beta =0$ where $\beta$ is a vector of regression coefficients, and $\text{L}$ is a design-like matrix specifying which elements in $\beta$ are being tested. The test statistic is computed using:

$${F}_{Wald}=\frac{{\left(\text{L}\widehat{\upbeta }\right)}^{t}{\left({\text{L}}^{t}\widehat{\text{V}}\text{L}\right)}^{-1}\left(\text{L}\widehat{\upbeta }\right)}{rank({\text{L}}^{t}\widehat{\text{V}}\text{L})}$$

Then, *p*-values are obtained from the F-distribution with numerator degrees of freedom equal to the number of tested parameters (or rank of ${\text{L}}^{t}\widehat{\text{V}}\text{L}$), and denominator degrees of freedom equal to the model degrees of freedom from the regression analysis minus the number of parameters tested (i.e., the sum of weights to approximate sample size minus the rank of ${\text{L}}^{t}\widehat{\text{V}}\text{L}$). In our case, the sample size for each country is usually large enough that the specific value for the denominator degrees of freedom is likely to have little bearing on the results. The above F-statistic is calculated for the resulting regression results from each imputed dataset. The F-statistic and degrees of freedom from each estimated test were saved and averaged across imputations. A global *p*-value is then obtained using the average F-values and degrees of freedom from the F-distribution ${p}_{global}\text{ } = 1-pF\left({F}_{pooled}|d{f}_{1,pooled}, d{f}_{2,pooled}\right),$ where $pF(.)$ is the cumulative F-distribution function, ${F}_{pooled}$ is the average F-statistic across imputed datasets, $d{f}_{1,pooled}$ and $d{f}_{2,pooled}$ are the average degrees of freedom across imputed datasets, and ${p}_{pooled}$ is the global *p*-value reported in each country-specific analysis.

The above F-test may have minor differences depending on software used, and the interested reader is referred to the package-specific documentation for joint tests: for R, see Lumley [33]; for Stata, see Stata Corp [29].test example 6; and for SAS, see SAS Institute [34] for tests with continuous outcome and see Padgett and Chen [31] for tests with binary outcomes.

#### E-values for sensitivity to unmeasured confounding and possible recall bias

For each childhood predictor, we calculated E-values to evaluate the sensitivity of results to potential unmeasured confounding. An E-value is the minimum strength of the association on the risk ratio scale that an unmeasured confounder must have with both the outcome and the predictor, above and beyond all measured covariates, for an unmeasured confounder to explain away an association [35]. A high E-value signifies that any unmeasured confounder would need to have a strong association with both the childhood predictor and the outcome to explain away the observed association. This suggests that the results are more likely to reflect a true causal relationship. An E-value closer to 1 signifies the opposite where the observed association may be explained away by an unmeasured confounder with a weak relationship with the outcome and predictor. Approximate E-values can be obtained for continuous outcomes through scale conversions [35]. E-values are provided for the country-specific results and for the meta-analysis estimated average effects—both random effects and population weighted estimates.

All of the childhood predictors are assessed retrospectively and are thus potentially subject to recall bias. The adult outcome may itself affect how participants recall their childhood experiences. This may be less likely for some childhood predictors (e.g., marital status of parents) than with others (e.g., self-rated health at age 12). Nevertheless, the concern needs to be taken into account in the interpretation of the analyses. It can, however, be shown that for recall bias to completely explain away the observed associations between the childhood predictors and the specific outcome would require that the effect of the adult outcome on biasing the retrospective assessments of the childhood predictor would essentially have to be at least as strong as the observed predictor-outcome associations themselves [36]. The observed multi-variable adjusted association itself thus constitutes an analogue of sort to the E-value for differential measurement error due to recall bias [36]. Comment will be made on this issue in the “Discussion” section within each of the individual childhood predictor manuscripts. In some cases, such as that illustrated below with mastery, recall bias might be sufficient to explain away some of the observed associations. However, when effect sizes are larger, this may be less plausible.

### Accounting for the complex sampling design

Accounting for the complex sampling design was accomplished by utilizing the information provided by Gallup on the primary sampling unit (PSU) IDs, strata IDs, and sampling weights. The weighting variable and PSU/strata IDs were included in all country-specific analyses. A complexity arises when respondents are recruited via face-to-face, because sometimes this results in groups (strata) with a single PSU. When a stratum has only a single case, this is known as a lonely PSU and makes variance and standard error estimation more complex because traditional methods assume multiple PSUs within each stratum [37, 38]. We elected to use the “certainty” specification where single-PSU stratum does not contribute to the variance; this maintained relatively comparable results across statistical software depending on the level of missingness in the childhood characteristic and on the specific outcome. Complete details concerning the implementation of these methods to account for the complex sampling design of each country can be found in the open code [39]. The methods were generally the same across all software packages, with very minor exceptions such as the singleton PSU issue mentioned above. We mostly relied on the default settings within each software package, which led to nearly identical results across software packages, with slight differences, principally in standard errors, mainly attributable to the imputation of missing data.

### Missing data and multiple imputation

All missing variables are imputed using multiple imputation by chained equations [40, 41]. The imputation model incorporated the criterion/outcome variable, all childhood/demographic characteristics, including race/ethnicity and childhood religious affiliation when available, and sampling weights. The sampling weights were included as a variable in the imputation models. Including the sampling weight in the multiple imputation procedure allowed study missingness to be related to the propensity of being included in the study. To avoid a singularity in the design matrix due to single-PSU strata, we elected not to include strata as a predictor in countries where strata were available. To account for variations in the assessment of certain variables across countries (e.g., race/ethnicity, childhood religious affiliation), we conducted the imputation process separately for each country. The within-country imputation approach ensured that the imputation model accurately reflects country-specific contexts and assessment methods.

When conducting multiple imputation, five imputed datasets are a commonly used default [42]. However, a more robust recommended number of imputations relate to the fraction of missing information (FMI) of the observed dataset [43]. The rate of missing data for this first wave of the GFS was quite low (< 5% for nearly all variables), and for the childhood predictor variables in particular, the item with the largest percent missing was the parent marital status variable (4.9%). Across all the items used as childhood predictors, the percent of respondents with any missing was 12.9% (a rough approximation of the FMI is therefore 0.129). Using an efficiency argument (FMI/m ≤ 0.05) commonly used, the number of imputed datasets needed would be ≈3. In preliminary testing, we evaluated using more imputed datasets (*m* = 20) and found no meaningful differences in results compared to only 5 imputations or when compared across software implementations of multiple imputation. Increasing past 5 imputed datasets was therefore thought to result in insufficient gains to justify the considerable increase in computational time due to the imputation being conducted separately by country and research team. However, we anticipate higher levels of missing data in subsequent waves due to wave-specific non-response, and analyses should consider using at least 20 imputations in subsequent waves of data analysis in spite of the additional computing time.

## Meta-analysis

The 22 countries were chosen to have broad geographical, cultural, and religious coverage; the countries include all six populated continents and represent about half of the world’s population. The random effects meta-analysis would be interpreted as estimating the pooled effect of each childhood predictor and the standard deviation of the childhood effect across countries from a hypothetical underlying population of which the sample of 22 countries would be representative. While such an underlying population is hypothetical, given the broad diverse coverage of the 22 countries, this was viewed as a reasonable target of interest. However, the results for each of the 22 countries are also provided, which are of interest in their own right, and may also be useful for readers who would prefer not to consider this underlying hypothetical population. Moreover, we provide a population-weighted fixed effects meta-analysis to evaluate similar childhood predictor effect where the principal target of inference concerns individual people in the 22 countries rather than the countries themselves.

All meta-analyses were conducted in R [20] using the *metafor* package [44] through an open-source application developed for these analyses [39]. The effect sizes, or values to be meta-analyzed, were the unstandardized regression coefficients and associated standard errors. We did not transform the country-specific regression coefficients.

### Random effects meta-analysis

For all the core GFS studies, a general random effects model was used, assuming the distribution of effect sizes in the population is normally distributed [45–47], that is:

$${y}_{i}\sim Normal({y}_{i}^{*},{v}_{i})$$

$${y}_{i}^{*}\sim Normal(\theta ,{\tau }^{2})$$

where ${y}_{i}$ is the unstandardized effect of the childhood predictor within each country, ${v}_{i}$ is the variance/uncertainty of ${y}_{i}$ within each country (i.e., the standard error of the regression coefficient), ${y}_{i}^{*}$ is the unknown “true effect” for the childhood predictor in country $i$, $\theta$ is the estimated average effect for the childhood predictor, and ${\tau }^{2}$ is the estimated variance/heterogeneity of ${y}_{i}^{*}$. The model was estimated using the Paule and Mandel estimator [48–50].

When a childhood predictor was highly collinear within a country, the resulting standard error of the effect can be large relative to the magnitude of the effect. Meta-analyzing several relatively imprecise effect estimates can result in the heterogeneity of effects to be severely underestimated (${\widehat{\tau }}^{2}<0.01$). A low estimate does not align with the mean differences in effects one sees when investigating the country-specific results, nor with a prior theoretical consideration. It is rather driven by the statistical artifact of extremely large standard errors arising from the multicollinearity of variables. The use of the Paule and Mandel estimator reduced the frequency of this occurring relative to restricted maximum likelihood in preliminary testing, but even the Paule and Mandel estimator did not eliminate the occurrence of this issue completely. In our results, we have noted when the estimates of heterogeneity based on the random effects meta-analyses are smaller than one would expect and refer readers to the accompanying online supplement where the forest plots provide a better indication of the heterogeneity (e.g., Q-statistics and Q-profile confidence intervals).

#### Proportion of effects outside a threshold

The output from the random effects meta-analyses includes the estimated proportion of effects estimates across countries with more substantial effect sizes lying above or below preregistered thresholds. These thresholds provide readers with an opportunity to gauge the distribution of effect sizes across countries in one particular manner, and are not intended to be used as strict benchmarks or cutoffs for determining whether observed associations are statistically or practically meaningful. For continuous outcomes, these thresholds were specified as unstandardized effects above 0.1, or below − 0.1. For binary outcomes, these thresholds were specified as risk ratios above 1.1, or below 0.9. Under substantial effect heterogeneity, there can in principal be notable proportions of effect in both directions. Estimates for the proportion of such effects were obtained using methodology based on calibrated effect sizes in meta-analyses The calibrated effect size is computed based on the meta-analysis results following well-established methods [51–53] that use the following formula:

$${\widetilde{y}}_{i}=\widehat{\theta }+\left({y}_{i}-\widehat{\theta }\right){\left(\frac{{\widehat{\tau }}^{2}}{{\widehat{\tau }}^{2}+{v}_{i}}\right)}^{0.5}$$

where ${\widetilde{y}}_{i}$ is the calibrated effect size of country $i$. The calibrated effect sizes were used to approximate the proportion of at least (or at most) a pre-specified bound. The proportion is approximated following Mathur and VanderWeele [52] by identifying the number of observed calibrated effect sizes and dividing them by the number of studies (e.g., 22).

For continuous outcomes, we approximated $Pr( {\widetilde{y}}_{i} < -0.10)$ and $Pr( {\widetilde{y}}_{i}> 0.10)$, where ${\widetilde{y}}_{i}$ is the calibrated unstandardized linear regression coefficient. For binary outcomes, we approximated $Pr(exp\left({\widetilde{y}}_{i}\right) < 0.90)$ and $Pr(exp( {\widetilde{y}}_{i})> 1.10)$, where $exp({\widetilde{y}}_{i})$ is the risk-ratio associated with effect ${\widetilde{y}}_{i}$. This *empirical* approach is the default method to approximate the proportion of effects by threshold [51]. However, another approximation is possible based on using the normal distribution, which aligns the estimation of proportions with the assumptions of the random effects meta-analysis. When the number of effects meta-analyzed is large, the meta-analytic mean ($\widehat{\theta }$) and standard deviation ($\widehat{\tau }$) can be used to estimate the proportion of effects that meet some specified lower threshold (${q}_{1}$) or upper threshold $({q}_{2})$, as follows:

$$\widehat{Pr}\left({y}_{i}<{q}_{1}\right)=\Phi \left(\frac{{q}_{1}-\widehat{\theta }}{\widehat{\tau }}\right)$$

$$\widehat{Pr}\left({y}_{i}>{q}_{2}\right)=1-\Phi \left(\frac{{q}_{2}-\widehat{\theta }}{\widehat{\tau }}\right)$$

where $\Phi \left(.\right)$ denotes the standard normal cumulative distribution function. This method is an option in our online app for meta-analysis but is not the default due to the relatively low number of countries (22) in the meta-analysis. In testing, we have found the proportions to be similar despite the relatively low number of effects being meta-analyzed.

### Population weighted meta-analysis

A fixed effects meta-analysis was conducted as a supplemental analysis to the random effects meta-analysis described above, providing an opportunity for researchers to consider both sets of results depending on which interpretative approach is most appropriate for their purposes. Inferences focused on differences across countries may utilize the random effects estimates, as these align with the target of inference, whereas analyses giving individuals equal weight align more with the results of the supplemental fixed effects meta-analyses. While the random effects meta-analysis assumes a distribution over the childhood predictors across countries (relaxing assumptions of measurement invariance somewhat), the supplemental fixed effects meta-analysis does not assume a distribution of effects but more directly estimates the weighted average effect over countries where the weight in this analysis is the total 2023 population (rather than the observed sample size) within each country. Note the fixed effects approach taken here essentially estimates the effect averaged across individuals in the various countries, and can be given this interpretation even if there is heterogeneity across countries in effect sizes [54]. The meta-analytic estimate is:

$$\widehat{\theta }=\frac{\sum {w}_{i}{y}_{i}}{\sum {w}_{i}}$$

where ${y}_{i}$ is the regression coefficient for each country and ${w}_{i}$ is the weight for each country. A common choice for the weight is the inverse of the sampling variance ${v}_{i}$, but in this analysis, we aimed to estimate the overall effect by treating individuals with equal weight instead of countries with equal weight, and without assuming a common effect size across countries. We therefore used a weight for each country that scales based on the total 2023 population size of each country.

Using the population sizes provided by Gallup, the fixed effects meta-analysis estimated the average effect of each childhood predictor weighted by the population size of each country. The country sizes used to create weights are shown in Table 1.

**Table 1:** Gallup provided estimates of population sizes (2023) for population weighted meta-analysis

Country	Population Est	Est. % of age 18 +	Est. population size of age 18 +	Meta-analysis weight
Argentina	35,576,161	0.930	33,085,830	0.014
Australia	21,255,952	0.960	20,329,009	0.008
Brazil	171,666,633	0.910	156,216,636	0.064
Egypt	74,517,704	0.750	56,059,669	0.023
Germany	72,343,802	0.960	69,392,175	0.028
Hong Kong (S.A.R of China)	6,461,584	0.940	6,097,316	0.003
India	1,058,538,924	0.910	964,761,394	0.395
Indonesia	206,057,132	0.940	193,828,725	0.079
Israel	6,869,826	0.900	6,191,774	0.003
Japan	110,582,052	0.970	107,139,250	0.044
Kenya	33,598,920	0.760	25,639,336	0.011
Mexico	96,256,568	0.930	89,518,608	0.037
Nigeria	124,471,001	0.940	117,351,260	0.048
Philippines	80,502,849	0.910	73,216,028	0.030
Poland	31,870,824	0.970	30,917,886	0.013
South Africa	42,793,704	0.910	38,728,302	0.016
Spain	41,045,172	0.950	39,144,781	0.016
Sweden	8,641,262	0.960	8,296,398	0.003
Tanzania	37,099,584	0.850	31,482,707	0.013
Turkey	65,514,213	0.960	62,703,653	0.026
United Kingdom	55,273,701	0.960	53,129,081	0.022
United States	273,430,984	0.950	259,759,435	0.106

*Note*. *S.A.R.* Special Administrative Region

### Global *p*-values (combining *p*-values from country-specific tests)

A decision was made to report *p*-values because it is common practice across many disciplines. The harmonic mean *p*-value was used to combine *p*-values across different countries [55, 56]. The combined *p*-value was used to test the null hypothesis of no effect of a variable (all categories have an estimated effect of 0 relative to baseline) in all countries, against the alternative hypothesis that in at least one country the group of regression coefficients (or risk ratios) for a given predictor are significantly different than 0 (or 1 for risk ratios). The harmonic mean *p*-value method is more robust to dependency among pooled *p*-values [56]. Although the country-specific tests are technically independent—an underlying assumption of most classic approaches to pooling *p*-values [57]—assuming independence of the *p*-values may not be entirely tenable given a common underlying set of items, translation procedures, childhood predictors, data cleaning techniques, and imputation models. To account for multiple testing, we present Bonferroni-corrected *p*-value significance thresholds for the meta-analytic results based on the number of predictors [58, 59] in the primary meta-analytic results. The Bonferroni adjustment for multiplicity was applied to the significance level cutoff (alpha) and not the *p*-values (we divided alpha by the number of tests and not multiplying the *p*-values by the number of tests). Providing the standard 0.05 significance threshold and Bonferroni-adjusted significance threshold provides transparency in how multiplicity was considered. However, the reported harmonic mean *p*-value is relatively robust to multiple testing already maintaining a constant type-I error rate regardless of the number of tests being conducted [56].

## Example analysis – sense of mastery

We will illustrate the aforementioned methodology and analyses and corresponding results with an example concerning childhood predictors of a person’s adult sense of mastery; see Kim et al. [13] for further details.

### Construct overview and importance

Sense of mastery—the perception that one has the ability to influence one’s environment and elicit desired outcomes [60, 61]—is important in its own right, but also because it shapes people’s trajectories of psychological, social, spiritual, behavioral, and physical health [62–66]. Sense of mastery was assessed in the GFS with the question, *How often do you feel very capable in most things you do in life?*; response options include always, often, rarely, and never, and was dichotomized by collapsing always/often and collapsing rarely/never.

### Illustrative results

Table 2 provides the distribution of nationally representative descriptive statistics of the demographic characteristics and candidate childhood predictors of the entire sample. Participant ages ranged the entire adult lifespan (18–80 +). Gender of the participants was nearly equally balanced between males (48%) and females (51%), with a small representation of other gender (0.3%). The majority of participants report either having a somewhat good or very good relationship with their mother (89%) and with their father (80%) while growing up. Attending religious services at least once a week at age 12 was reported by an estimated 41% of participants.

**Table 2:** Nationally representative descriptive statistics of the observed sample

**Characteristic**	***N* = 202,898**^a^
**Relationship with mother**	
Very good	127,836 (63%)
Somewhat good	52,439 (26%)
Somewhat bad	11,060 (5.5%)
Very bad	4,642 (2.3%)
Does not apply	5,965 (2.9%)
(Missing)	956 (0.5%)
**Relationship with father**	
Very good	107,742 (53%)
Somewhat good	55,714 (27%)
Somewhat bad	15,807 (7.8%)
Very bad	8,278 (4.1%)
Does not apply	13,985 (6.9%)
(Missing)	1,372 (0.7%)
**Parent marital status**	
Parents married	152,001 (75%)
Divorced	17,726 (8.7%)
Parents were never married	15,534 (7.7%)
One or both parents had died	7,794 (3.8%)
(Missing)	9,843 (4.9%)
**Subjective financial status of family growing up**	
Lived comfortably	70,861 (35%)
Got by	82,905 (41%)
Found it difficult	35,852 (18%)
Found it very difficult	12,606 (6.2%)
(Missing)	674 (0.3%)
**Abuse**	
Yes	29,139 (14%)
No	167,279 (82%)
(Missing)	6,479 (3.2%)
**Outsider growing up**	
Yes	28,732 (14%)
No	170,577 (84%)
(Missing)	3,589 (1.8%)
**Self-rated health growing up**	
Excellent	67,121 (33%)
Very good	63,086 (31%)
Good	47,378 (23%)
Fair	19,877 (9.8%)
Poor	4,906 (2.4%)
(Missing)	530 (0.3%)
**Immigration status**	
Born in this country	190,998 (94%)
Born in another country	9,791 (4.8%)
(Missing)	2110 (1.0%)
**Age 12 religious service attendance**	
At least 1/week	83,237 (41%)
1–3/month	33,308 (16%)
< 1/month	36,928 (18%)
Never	47,445 (23%)
(Missing)	1,980 (1.0%)
**Year of birth**	
1998–2005; current age 18–24	27,007 (13%)
1993–1998; age 25–29	20,700 (10%)
1983–1993; age 30–39	40,256 (20%)
1973–1983; age 40–49	34,464 (17%)
1963–1973; age 50–59	31,793 (16%)
1953–1963; age 60–69	27,763 (14%)
1943–1953; age 70–79	16,776 (8.3%)
1943 or earlier; age 80 +	4119 (2.0%)
(Missing)	20 (< 0.1%)
**Gender**	
Male	98,411 (49%)
Female	103,488 (51%)
Other	602 (0.3%)
(Missing)	397 (0.2%)
**Country**	
Argentina	6724 (3.3%)
Australia	3844 (1.9%)
Brazil	13,204 (6.5%)
Egypt	4729 (2.3%)
Germany	9506 (4.7%)
Hong Kong (S.A.R. of China)	3012 (1.5%)
India	12,765 (6.3%)
Indonesia	6992 (3.4%)
Israel	3669 (1.8%)
Japan	20,543 (10%)
Kenya	11,389 (5.6%)
Mexico	5776 (2.8%)
Nigeria	6827 (3.4%)
Philippines	5292 (2.6%)
Poland	10,389 (5.1%)
South Africa	2651 (1.3%)
Spain	6290 (3.1%)
Sweden	15,068 (7.4%)
Tanzania	9075 (4.5%)
Turkey	1473 (0.7%)
United Kingdom	5368 (2.6%)
United States	38,312 (19%)

*Note*. *S.A.R.* Special Administrative Region

^a^Weighted statistics computed using the tbl_svysummary function of the *gtsummary* package in R [20]

Table 3 provides the meta-analytic estimates of the effects of childhood experiences on a sense of mastery. A similar table is presented in all construct-specific manuscripts that include childhood predictor analyses following the template reported in this article. No single childhood predictor appeared to dominantly predict sense of mastery in adulthood; instead, a combination of several childhood predictors appears to be at play. For this particular analysis, most effect sizes were small and the estimated proportions with risk ratios above 1.1 or below 0.9 were likewise quite small. This was not the case with some other outcomes in the GFS, however. The results here are given for illustrative purposes. See Kim et al. [13] for further substantive interpretation.

**Table 3:** Random effects meta-analysis of regression of sense of mastery on childhood predictors

				Estimated proportion of effects by threshold			
Variable	Category	RR^1^	95% CI	< 0.90	> 1.10	*I*^2^	Global *p*-value
Relationship with mother	(Ref: very bad/somewhat bad)						0.016*
	Very good/somewhat good	1.03	(1.01, 1.06)	0.00	0.05	57.5	
Relationship with father	(Ref: very bad/somewhat bad)						0.007*
	Very good/somewhat good	1.02	(1.00, 1.03)	0.00	0.00	41.7	
Parent marital status	(Ref: parents married)						< .001**
	No, divorced	0.99	(0.97, 1.01)	0.00	0.00	32.5	
	Single, never married	1.01	(0.97, 1.05)	0.05	0.18	90.9	
	No, one, or both had died	0.98	(0.94, 1.01)	0.14	0.05	72.1	
Subjective financial status of family growing up	(Ref: got by)						< .001**
	Lived comfortably	1.03	(1.01, 1.05)	0.00	0.05	81.3	
	Found it difficult	0.98	(0.97, 0.99)	0.00	0.00	23.5	
	Found it very difficult	0.96	(0.93, 0.98)	0.05	0.00	42.6	
Abuse	(Ref: no)						< .001**
	Yes	0.96	(0.94, 0.98)	0.10	0.00	79.5	
Outsider growing up	(Ref: no)						< .001**
	Yes	0.95	(0.93, 0.97)	0.09	0.00	63.0	
Self-rated health growing up	(Ref: good)						< 0.001**
	Excellent	1.08	(1.04, 1.11)	0.00	0.18	92.3	
	Very good	1.04	(1.02, 1.06)	0.00	0.09	77.0	
	Fair	0.94	(0.92, 0.97)	0.27	0.00	67.9	
	Poor	0.93	(0.89, 0.97)	0.23	0.00	45.9	
Immigration status	(Ref: born in this country)						< 0.001**
	No	1.02	(0.99, 1.06)	0.00	0.14	78.6	
Age 12 religious service attendance	(Ref: never)						< 0.001**
	At least 1/week	1.05	(1.03, 1.08)	0.00	0.14	72.3	
	1–3/month	1.05	(1.01, 1.09)	0.00	0.09	89.9	
	Less than 1/month	1.03	(1.01, 1.04)	0.00	0.05	56.4	
Year of birth	(Ref: 1998–2005; age 18–24)						< 0.001**
	1993–1998; age 25–29	1.01	(0.99, 1.03)	0.00	0.05	63.7	
	1983–1993; age 30–39	1.03	(1.00, 1.07)	0.00	0.23	88.9	
	1973–1983; age 40–49	1.03	(0.98, 1.07)	0.14	0.23	92.2	
	1963–1973; age 50–59	1.04	(0.99, 1.09)	0.18	0.27	93.6	
	1953–1963; age 60–69	1.04	(0.99, 1.10)	0.09	0.36	92.2	
	1943–1953; age 70–79	1.05	(0.99, 1.12)	0.14	0.32	90.2	
	1943 or earlier; age 80 + ^a^	1.07	(0.98, 1.16)	0.27	0.50	89.9	
Gender	(Ref: male)						< 0.001**
	Female	0.98	(0.97, 0.99)	0.00	0.00	60.4	
	Other^a^	0.47	(0.13, 1.65)	0.56	0.28	99.9	

Note. *N* = 202,898

^*^p < 0.05

^**^p < 0.004 (Bonferroni-corrected threshold)

^a^Group is very small (< 0.1% of the observed sample) within several countries, leading to complete separation and large uncertainty in this estimate—be cautious about interpreting this estimate

^1^*RR* = risk ratio, the RR is interpreted, for example—the RR for gender female, as the ratio of the proportion of females who endorsed often/always to feeling a sense of mastery relative the proportion of males who endorsed often/always to feeling a sense of mastery; *CI* confidence interval; the estimated proportion of effects is the estimated proportion of RR above (or below) a threshold based on the calibrated effect sizes [52]; *I*^2^ is an estimate of the variability in means due to heterogeneity across countries vs. sampling variability; the global *p*-value corresponds to the joint test of the null hypothesis that the country-specific joint parameter Wald tests (all parameters within variable groups are zero) are all null all 22 countries; and additional details of heterogeneity of effects—including estimates of heterogeneity ($\tau$) on the log-risk scale—are available in the forest plots

Table 4 provides E-value estimates for the meta-analysis. E-values indicated that some of the observed associations were potentially slightly to moderately robust to unmeasured confounding (Table 4). For example, when considering relationship with mother, an unmeasured confounder that was associated with both mastery and relationship with mother by risk ratios of 1.22 each (above and beyond the covariates already adjusted for) could explain away the association, but weaker joint confounder associations could not. Furthermore, to shift the CI to include the null, an unmeasured confounder associated with both sense of control and relationship with mother by risk ratios of 1.09 each could explain away the association, but weaker joint confounder associations could not. In several cases, a combination of unmeasured confounding and statistical uncertainty might suffice to explain away the results; and likewise, differential measurement error due to recall bias might suffice to explain away some of the results [35] given the modest effect sizes in Table 3.

**Table 4:** Sensitivity of meta-analyzed childhood predictors to unmeasured confounding

Variable	Category	E-value for estimate	E-value for 95% CI
Relationship with mother	(Ref: very bad/somewhat bad)		
	Very good/somewhat good	1.22	1.09
Relationship with father	(Ref: very bad/somewhat bad)		
	Very good/somewhat good	1.15	1.03
Parent marital status	(Ref: parents married)		
	No, divorced	1.12	1.00
	Single, never married	1.11	1.00
	No, one or both had died	1.18	1.00
Subjective financial status of family growing up	(Ref: got by)		
	Lived comfortably	1.20	1.13
	Found it difficult	1.16	1.09
	Found it very difficult	1.26	1.16
Abuse	(Ref: no)		
	Yes	1.25	1.14
Outsider growing up	(Ref: no)		
	Yes	1.28	1.21
Self-rated health growing up	(Ref: good)		
	Excellent	1.37	1.25
	Very good	1.26	1.18
	Fair	1.32	1.22
	Poor	1.36	1.21
Immigration status	(Ref: born in this country)		
	Born in another country	1.17	1.00
Age 12 religious service attendance	(Ref: never)		
	At least 1/week	1.29	1.20
	1–3/month	1.29	1.13
	Less than 1/month	1.19	1.11
Year of birth	(Ref: 1998–2005; age 18–24)		
	1993–1998; age 25–29	1.12	1.00
	1983–1993; age 30–39	1.21	1.00
	1973–1983; age 40–49	1.19	1.00
	1963–1973; age 50–59	1.25	1.00
	1953–1963; age 60–69	1.25	1.00
	1943–1953; age 70–79	1.28	1.00
	1943 or earlier; age 80 + ^a^	1.34	1.00
Gender	(Ref: Male)		
	Female	1.18	1.13
	Other^a^	3.70	1.00

Note. *N* = 202,898; the E-value is the minimum strength of the association an unmeasured confounder must have with both the outcome (sense of mastery) and the predictor, above and beyond all measured covariates, for an unmeasured confounder to explain away an association [36]; and ^a^Group is very small (< 0.1% of the observed sample) within several countries potentially leading to complete separation and large uncertainty in this estimate—be cautious about interpreting this estimate

**Fig. 1:**
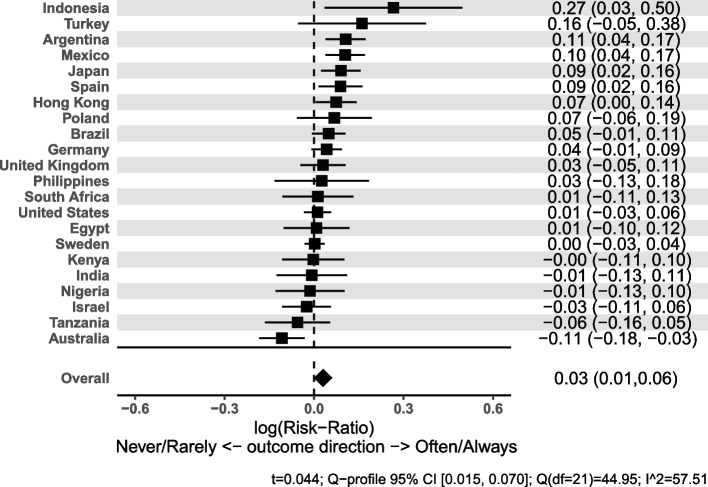
Forest plot for the meta-analysis of the effect of a “Very good/somewhat good” relationship with mother compared to a “Very bad/somewhat bad” relationship on sense of mastery

The online supplemental material in each manuscript will have a corresponding forest plot for each childhood predictor category included in the meta-analysis, which displays the meta-analyzed regression coefficients, the country-specific effect estimates, and additional information on heterogeneity of effects. An example forest plot for the meta-analysis of regression coefficients for sense of mastery across countries for the effect of a “Very good/somewhat good” relationship with mother category is shown in Fig. 1. The forest plots are constructed such that all effects are ordered by magnitude and the *y*-axis varies, allowing for a quick inspection of which countries have a high or low effect and whether these orders are similar across effects. The country-specific estimates reported in the online supplemental tables of each manuscript provide a complementary view that compares the magnitude of effects across predictors within a country, in contrast to the forest plots, which provide a comparison across countries for a predictor category (relative to the reference category). We repeated the meta-analyses using a population weighted (fixed effects) approach to estimate effects if we were to weight within-country results by the size of the population the sample represents. Those results are not shown here but are shown in the online supplemental material of each individual manuscript.

## Strengths and limitations

The analytic methodology employed for the study of childhood predictors of outcomes related to well-being has several strengths and limitations. A notable strength is the broad population coverage of the GFS. The countries included in wave 1 of the GFS encompasses approximately 64% of the world’s population [3]. Most of the analytic methods employed are relatively well-established methods with a long history of being used in either epidemiology, public health, psychology, or sociology research. We aimed to employ rigorous methods that appropriately incorporate the unique complex sampling design used in each country to obtain robust standard errors. In line with this aim, we implemented macros and packages as needed [31]. All code to reproduce analyses are openly available in several languages (R, SAS, SPSS, and Stata) for researchers to explore these data and results. All analyses were conducted at the country-level, and the results were then pooled using meta-analytic techniques to account for uncertainty in the estimates and quantify heterogeneity across countries. We used a random effects “distribution of effects” perspective of meta-analytic methods for our primary analyses, but also reported a fixed effects “population-weighted” perspective as a supplemental analysis. Using different theoretical perspectives for pooling estimates provides flexibility to the reader to interpret which set of effects are appropriate for their purposes.

There are limitations to consider as well. Sources of heterogeneity in the relationship between childhood predictors and outcomes across countries could be due to seasonality effects, differences in interpretation, differences due to quality of translation, differences in mode of data collection, differences in the process and variables used for constructing respondent level weights, and other possible reasons depending on the specific construct of interest [15]. Most of the psychosocial constructs that were assessed only had a single item to represent the overall construct (e.g., sense of mastery), many of which were assessed with binary or ordinal response scales with few categories. Although it is not uncommon for such items to be used in large-scale epidemiologic studies such as the GFS and decisions about which items and response scales to use were guided by several phases of GFS survey development [12], some measures in the GFS survey may not be a suitable fit for answering certain research questions. The use of single-item assessments provides less construct coverage and generally lower true-score reliability, resulting in less power to detect effects of the candidate childhood predictors on well-being outcomes [67]. The anticipated effect sizes of the childhood predictors using a retrospective recall approach were small, leading to the need to have large sample sizes to detect these small effects. The obtained country sample sizes for wave 1 were relatively large (ranging from ~ 1500 to 38,000), which helps to identify small effects anticipated.

The use of retrospective recall is not without limitations and issues as well. The retrospective assessment of the childhood predictors allowed for a synthetic longitudinal design to be constructed using wave 1 of the GFS. The findings from these analyses may be influenced by recall bias [68] and common method bias [69]. To mitigate these sources of bias, future research might employ prospective longitudinal designs that follow individuals from childhood to adulthood and complement self-report survey responses with data derived from other sources (e.g., parents). One side effect of using a retrospective recall approach to artificially construct a longitudinal analysis is that the recalled characteristics can be highly correlated. It would be reasonable to expect one’s perceptions of their health growing up to be related to their family’s financial status growing up or that their perspectives on relationship with their parent will be related to how much love they feel from their parent. These limitations led to issues with inclusion of all initially planned childhood predictors, which was addressed using the approach described in the “ Childhood predictor variables” section.

Several limitations on the side of the statistical methods employed are noted next. The regression analyses used a common form (see the “ Regression analyses” section) that may not be optimal for all outcomes. For example, the illustrative analysis of regression sense of mastery on all childhood predictors assumes differences in the log(risk-ratio) of endorsing always/often over rarely/never can be modeled as a linear combination of all childhood predictors. Exploratory analyses to evaluate potential nonlinearities in this relationship could be valuable for each outcome in future works. Additionally, the precise implementations of the methods to account for the complex sampling design can sometimes be not fully transparent, especially in software packages that require a license (e.g., SAS, Stata). The use of several software packages helped to identify the effects of any software-specific peculiarities. A common issue we needed to deal with involved handling “lonely PSUs” [38], but we aimed to always use a “certainty” specification that fixed the variance contribution to zero in such cases when estimating variance components. This approach has the limitation of potentially underestimating the variance, or standard error, for a particular estimate. However, to the best of our knowledge, there is no generally agreed upon approach for handling such instances, and our aim is to be transparent about these decisions to reduce non-reproducibility because of unclear analytic decisions and researcher degrees of freedom [70].

The analyses outlined in this article are relatively straightforward, but also varied to allow for multiple interpretive lenses to be applied (e.g., within-country vs. cross-country patterns). Implementing these coordinated analyses has its challenges, such as complications implementing analyses using complex sampling weights, multiple imputation, modified Poisson regression, and meta-analysis across several statistical packages, and yet we found remarkably similar results across packages in spite of slightly different implementations [24].

## Conclusions

The current article provides a description of the methods used in manuscripts reporting childhood predictors analyses that leverage currently available wave 1 data from the GFS, most of which are being considered for publication as a coordinated set of manuscripts based on the GFS. Using nationally representative data from 22 geographically and culturally diverse countries around the world, the current set of planned childhood predictors analyses provide a unique opportunity to (1) explore the relationship between childhood experiences and many construct indicators related to subsequent adult well-being and (2) identify potentially modifiable childhood factors that could be targeted through intervention to support population-level well-being. The interested reader is referred to our companion article, *Analytic Methodology for Demographic Variation Analyses for Wave 1 of the Global Flourishing Study* [71], for a description of the methods used in the coordinated set of demographic variation analyses of GFS outcomes.

## Supplementary Information

Additional file 1: Table S1 that is referenced as supporting information of our process of examining multicollinearity

## Data Availability

Data for Wave 1 of the GFS is available through the Center for Open Science (https://www.cos.io/gfs) upon submission of a pre-registration, and will be openly available without pre-registration beginning February 2025. Subsequent waves of the GFS will similarly be made available. Please see https://www.cos.io/gfs-access-data for more information about data access. Code for the GFS childhood predictor analyses in multiple software is openly available (10.17605/osf.io/vbype).
