# Supplementary material for: Analytic methodology for childhood predictor analyses for wave 1 of the Global Flourishing Study
Source: BMC Glob Public Health. 2025 Apr 30;3:29. doi: 10.1186/s44263-025-00142-0 (PMC12042376; doi:10.1186/s44263-025-00142-0)
Supplement: Supplementary file 1 — Additional file 1: Table S1 that is referenced as supporting information of our process of examining multicollinearity [file 44263_2025_142_MOESM1_ESM.docx]

**Additional file 1**

| **Table S1**.  *Initially pre-registered childhood predictors were prone to multicollinearity issues—estimates shown are the unstandardized effects of each childhood predictors on the log-risk scale.* | | | | | | | | | | |
| --- | --- | --- | --- | --- | --- | --- | --- | --- | --- | --- |
|  |  | Turkey (N=1473) | |  | United States  (N=38,312) | |  | Meta-analysis  (N=202,898) | | |
| Variable | Category | Est | SE |  | Est | SE |  | Est | SE | p |
| Age 12 religious | (Ref: Never) |  |  |  |  |  |  |  |  |  |
| service attendance | At least 1/week | 0.06 | 0.06 |  | 0.03 | 0.02 |  | 0.03 | 0.01 | <0.01 |
|  | 1-3/month | 0.06 | 0.07 |  | 0.05 | 0.02 |  | 0.03 | 0.02 | 0.06 |
|  | < 1/month | -0.02 | 0.07 |  | 0.01 | 0.03 |  | 0.02 | 0.01 | 0.02 |
| Religious service | (Ref: Never) |  |  |  |  |  |  |  |  |  |
| attendance of | At least 1/week | 0.01 | 0.06 |  | -0.02 | 0.02 |  | 0.02 | 0.01 | 0.02 |
| father | **1-3/month** | **-0.07** | **0.07** |  | **-0.03** | **0.02** |  | **0.01** | **0.01** | **0.03** |
|  | <1/month | -0.09 | 0.09 |  | -0.01 | 0.02 |  | 0.01 | 0.01 | <0.01 |
| Religious service | (Ref: Never) |  |  |  |  |  |  |  |  |  |
| attendance of | At least 1/week | 0.09 | 0.06 |  | -0.01 | 0.02 |  | 0.01 | 0.01 | 0.03 |
| mother | **1-3/month** | **0.07** | **0.06** |  | **-0.01** | **0.02** |  | **0.01** | **0.01** | **0.01** |
|  | <1/month | 0.10 | 0.07 |  | 0.00 | 0.02 |  | 0.01 | 0.01 | 0.02 |
|  |  |  |  |  |  |  |  |  |  |  |
| Love from father | (Ref: No) |  |  |  |  |  |  |  |  |  |
|  | Yes | -0.01 | 0.07 |  | 0.04 | 0.03 |  | 0.03 | 0.01 | <0.01 |
| Relationship with father | (Ref: Very bad) |  |  |  |  |  |  |  |  |  |
|  | Somewhat bad | 0.02 | 0.10 |  | 0.01 | 0.04 |  | -0.01 | 0.01 | <0.01 |
|  | Somewhat good | -0.03 | 0.10 |  | -0.02 | 0.04 |  | -0.02 | 0.01 | <0.01 |
|  | Very good | -0.11 | 0.10 |  | -0.01 | 0.04 |  | 0.00 | 0.01 | <0.01 |
| Love from mother | (Ref: No) |  |  |  |  |  |  |  |  |  |
|  | Yes | 0.05 | 0.10 |  | 0.00 | 0.03 |  | 0.01 | 0.01 | 0.04 |
| Relationship with mother | (Ref: Very bad) |  |  |  |  |  |  |  |  |  |
|  | Somewhat bad | -0.36 | 0.20 |  | 0.00 | 0.05 |  | -0.02 | 0.02 | <0.01 |
|  | Somewhat good | -0.06 | 0.15 |  | 0.01 | 0.05 |  | 0.01 | 0.02 | 0.06 |
|  | Very good | -0.02 | 0.15 |  | 0.00 | 0.05 |  | 0.02 | 0.02 | 0.06 |
| Parent marital status | (Ref: Parents married) |  |  |  |  |  |  |  |  |  |
|  | Divorced | -0.11 | 0.11 |  | 0.02 | 0.02 |  | -0.01 | 0.01 | 0.02 |
|  | One or both parents had died | -0.01 | 0.12 |  | -0.01 | 0.04 |  | -0.02 | 0.02 | 0.06 |
|  | Single, never married | 0.30 | 0.08 |  | -0.04 | 0.04 |  | 0.01 | 0.02 | 0.09 |
|  |  |  |  |  |  |  |  |  |  |  |
| Self-rated health | (Ref: Good) |  |  |  |  |  |  |  |  |  |
| growing up | Excellent | 0.07 | 0.05 |  | 0.09 | 0.02 |  | 0.07 | 0.02 | 0.07 |
|  | Very good | 0.04 | 0.05 |  | 0.05 | 0.02 |  | 0.04 | 0.01 | 0.04 |
|  | Fair | -0.11 | 0.07 |  | -0.14 | 0.05 |  | -0.06 | 0.01 | 0.05 |
|  | Poor | -0.20 | 0.15 |  | -0.18 | 0.10 |  | -0.07 | 0.02 | 0.07 |
|  |  |  |  |  |  |  |  |  |  |  |
| Subjective financial | (Ref: Got by) |  |  |  |  |  |  |  |  |  |
| status of family | Found it difficult | -0.10 | 0.06 |  | 0.00 | 0.02 |  | -0.04 | 0.01 | 0.01 |
| growing up | Found it very difficult | -0.09 | 0.10 |  | -0.03 | 0.04 |  | -0.02 | 0.01 | 0.03 |
|  | Lived comfortably | 0.01 | 0.04 |  | 0.03 | 0.01 |  | 0.03 | 0.01 | 0.03 |
|  |  |  |  |  |  |  |  |  |  |  |
| Abuse | (Ref: No) |  |  |  |  |  |  |  |  |  |
|  | Yes | 0.05 | 0.06 |  | -0.02 | 0.02 |  | -0.04 | 0.01 | 0.05 |
| Outsider growing up | (Ref: No) |  |  |  |  |  |  |  |  |  |
|  | Yes | -0.01 | 0.06 |  | -0.12 | 0.02 |  | -0.04 | 0.01 | 0.04 |
| Immigration status | (Ref: Born in this country) |  |  |  |  |  |  |  |  |  |
|  | Born in another country | -0.07 | 0.12 |  | 0.00 | 0.03 |  | 0.03 | 0.02 | 0.06 |
| Year of birth | (Ref: 1998-2005; age: 18-24) |  |  |  |  |  |  |  |  |  |
|  | 1993-1998; age 25-29 | 0.05 | 0.07 |  | -0.04 | 0.06 |  | 0.01 | 0.01 | 0.04 |
|  | 1983-1993; age 30-39 | 0.18 | 0.06 |  | 0.08 | 0.05 |  | 0.03 | 0.02 | 0.07 |
|  | 1973-1983; age 40-49 | 0.06 | 0.06 |  | 0.11 | 0.05 |  | 0.03 | 0.02 | 0.10 |
|  | 1963-1973; age 50-59 | 0.16 | 0.06 |  | 0.14 | 0.05 |  | 0.04 | 0.03 | 0.11 |
|  | 1953-1963; age 60-69 | 0.03 | 0.09 |  | 0.16 | 0.05 |  | 0.04 | 0.03 | 0.12 |
|  | 1943-1953; age 70-79 | -0.05 | 0.16 |  | 0.17 | 0.05 |  | 0.05 | 0.03 | 0.13 |
|  | 1943 or earlier; age 80+ | 0.12 | 0.19 |  | 0.15 | 0.05 |  | 0.06 | 0.04 | 0.18 |
| Gender | (Ref: Male) |  |  |  |  |  |  |  |  |  |
|  | Female | 0.02 | 0.04 |  | 0.00 | 0.01 |  | -0.02 | 0.00 | 0.02 |
|  | Other | * | * |  | 0.01 | 0.07 |  | -0.73 | 0.64 | 2.70 |
| *Note*. The bolded rows of religious service attendance frequency for mother and father while growing up provides an example of multicollinearity where the effects cancel out leading to uninterpretable estimates. *Effect not estimable due to no cases endorsing ‘other’ for Gender. | | | | | | | | | | |
